# Effects of Different Die Metals on the Performance and Friction and Wear of Composite Materials during the Extrusion Process

**DOI:** 10.3390/polym15244684

**Published:** 2023-12-12

**Authors:** Hong Liu, Chuansheng Wang

**Affiliations:** 1 College of Electromechanical Engineering, Qingdao University of Science and Technology, Qingdao 266061, China; liuhongbb@126.com; 2 National Engineering Laboratory for Advanced Tire Equipment and Key Materials, Qingdao 266061, China

**Keywords:** extruder head, 38CrMoAlA, Hastelloy C-276, wear, rubber properties

## Abstract

Extrusion technology is widely utilized in the rubber processing industry, with the extruder serving as the core equipment. As mixed rubber enters the extruder, it undergoes conveyance and plasticization, ultimately forming specific shapes and dimensions upon extrusion. The extruder head is a crucial component, playing a key role in achieving the final product’s required size and shape. Factors such as its structure, materials, and manufacturing processes significantly impact the efficiency, product quality, and sustainability of the extrusion process. However, prolonged operation leads to severe wear of the extruder head, adversely affecting rubber product quality. Additionally, extruder head processing poses challenges, with maintenance and repair being complex procedures. Therefore, exploring a wear-resistant, long-lasting metal material for the extruder head without compromising mixed rubber performance is essential. This study focuses on severely worn extruder head metal materials, comparing wear levels after friction with STELLITE 6 alloy, Hastelloy C-276 alloy, 38CrMoAlA, and tungsten carbide with composite rubber. Results show that compared to the NR/BR composite material after Hastelloy C-276 alloy friction, rubber Payne effect increased by 4.4% (38CrMoAl), 3.2% (STELLITE 6), and 4.6% (tungsten carbide). Similarly, rubber dispersion decreased by 9.4% (38CrMoAl), 4.7% (STELLITE 6), and 9.8% (tungsten carbide). Rolling resistance increased by 18.1% (38CrMoAl), 16% (STELLITE 6), and 23.4% (tungsten carbide). Friction coefficient increased by 3.5% (38CrMoAl), 2.8% (STELLITE 6), and 4.3% (tungsten carbide). Wear volume increased by 39.3% (38CrMoAl), 45.3% (STELLITE 6), and 48.9% (tungsten carbide). Specifically, using Hastelloy C-276 alloy as the extruder head metal material yields the best NR/BR composite material dispersion, highest ten times tear strength, excellent anti-wet skid resistance, and minimum rolling resistance. Conversely, using the other alloys results in varying reductions in the physical and mechanical properties of NR/BR composite materials. This research is crucial for improving rubber product quality and extending extruder head lifespan.

## 1. Introduction

Extrusion technology represents a widely utilized processing method in the rubber industry. The extruder machine stands as a pivotal apparatus for realizing the extrusion process, where compounded rubber enters, undergoes plasticization through the feeding, plasticizing, and extrusion sections, and is ultimately extruded into products of specified shapes and dimensions. Among these components, the extruder head, as a key element of the extruder, plays a crucial role in achieving the final product’s desired shape and size. Factors encompassing its structure, materials, and manufacturing processes significantly impact the efficiency, product quality, and sustainability of the extrusion process. This paper aims to provide an overview of the current research status on extruder heads, encompassing their structure, manufacturing processes, and future development directions.

Throughout the entire extrusion process, compounded rubber is pressurized, flows through the extruder screw and barrel, and reaches the extruder head, where it is ultimately shaped into the desired dimensions through the head’s channel. During this process, the compounded rubber comes into direct contact with the interior surface of the extruder head. In practical production, the interior surface of the extruder head undergoes frictional wear after a period of use, which consequently affects the dimensions and shapes of the extruded products, as well as the various properties of the compounded rubber.

Yuzhen Yu et al. [1,2,3,4,5] conducted research on pipe extruder heads, elucidating their structure and operational principles. They delineated the current status and trends in pipe extruder head development, providing valuable insights for future research. They also established mathematical models for the extrusion process and employed Polyflow simulation software to study the flow field within the extruder head channel, thereby offering a theoretical foundation for the structural design and optimization of head channels.

Liu Huan [6] conducted experiments with a self-designed high-shear extruder head for the extrusion of low-density polyethylene/polyethylene terephthalate (LDPE/PET) blends. They employed scanning electron microscopy (SEM) to observe the microstructure of the extruded products and investigated the in situ formation of microfibers during the extrusion process. Results indicated that increasing the PET phase, reducing the gap in the high-shear extruder head, elevating rotor speed, and enhancing the pull-cutting speed led to decreased melt flow rates (MFRs) of the extruded products. SEM analysis of the fractured surfaces of the extruded products revealed the formation of numerous microfibers in the blend, with an average diameter of 3–5 μm and an aspect ratio exceeding 10.

To mitigate the adverse effects of uneven pressure distribution on hose quality, Xiaodong Lv [7] optimized the design of the extruder head for the outer layer of hoses. This optimization involved adding an inner liner to the extruder head’s rear cover to prevent the most uneven pressure distribution segment from directly impacting the hose. Additionally, a ring-shaped material storage cavity was incorporated into the head to ensure even material flow into the die from all directions, effectively eliminating pressure-related variations in the outer layer’s thickness. Finite element analysis was performed on the designed head using ANSYS/Polyflow software to assess its feasibility.

Guangyi Lin et al. [8,9,10,11,12] developed different three-dimensional physical models for L-type sheet extruder heads and employed software such as ANSYS WORKBENCH and POLYFLOW to simulate the pressure and velocity fields of the head’s internal fluid, thereby analyzing the conditions for achieving effective extrusion.

Li Li et al. [13,14,15,16,17,18,19,20] investigated the extrusion of rubber composite materials using various head configurations, including stacked orientation heads and opposed heads, under identical experimental conditions involving head pressure, head temperature, and screw speed. They conducted comparative analyses of the pressure fields within the flow channels of different head types and standard extruder heads, ensuring improved extrusion and product quality of reinforced rubber composite materials.

The extruder head of the extrusion machine is a critical component of extrusion technology, with its structure, materials, and manufacturing processes significantly influencing the efficiency and product quality of the extrusion process [21,22,23,24,25,26,27,28,29,30]. The damage evolution of powder metallurgy copper/titanium dioxide composites, with a focus on the reduction of Young’s modulus, was investigated by Majzoobi GH [31]. The study revealed that the effective parameters influencing damage evolution include strain rate, plastic strain, fracture strain, reinforced particle volume fraction, and particle aspect ratio. These parameters were incorporated into a novel model employed for predicting the damage evolution of metal matrix composites (MMCs). The validation of this model was conducted through experimental results. The selection of materials for extruder heads necessitates consideration of mechanical properties, thermal characteristics, corrosion resistance, thermal conductivity, and cost, among other factors. Common materials for extruder heads include cast steel, 42CrMo alloy steel, and 38CrMoAlA. Furthermore, surface treatment techniques such as nitriding and hard chromium plating can enhance head performance. With the ongoing development of materials science, researchers are exploring new materials for extruder heads to improve their wear resistance and corrosion resistance. High-performance ceramics, composite materials, and high-temperature, high-pressure alloys are being considered for extruder heads to enhance their durability and performance. Future research and development in extruder heads will focus on improving their performance, reducing energy consumption, and minimizing environmental impact [32,33,34,35,36,37,38,39,40,41]. In conclusion, the study and development of extruder heads are crucial for enhancing production efficiency, product quality, and sustainability. Future research will address areas such as intelligence, sustainability, energy efficiency, new material applications, and flexible customization to meet evolving market demands and environmental requirements. International collaboration and knowledge sharing will drive continuous progress and innovation in the field of extruder heads.

While scholars worldwide have researched extruder head friction [42,43,44,45,46,47,48,49,50,51], prior studies have primarily focused on commonly used cast steel materials and have not considered the impact of worn metal particles on the properties of compounded rubber. This paper, considering practical operating conditions, investigates four extruder head metal materials: 38CrMoAlA, STELLITE 6 alloy, Hastelloy C-276 alloy, and carbide-coated metals.

## 2. Experiments

### 2.1. Experimental Equipment

This equipment list mentions various instruments used in the field of material testing and analysis:

1. Cold feed pin extruder. Manufacturer: Products of Qingdao University of Science and Technology, Qingdao, China.
2. LEXT OLS5000 3D Laser Measurement Microscope. Manufacturer: Olympus Corporation, Tokyo, Japan.
3. DisperGRADER Dispersion Analyzer. Manufacturer: Alpha Corporation, Dayton, OH, USA.
4. RPA2000 Rubber Processing Performance Analyzer. Manufacturer: Alpha Corporation, Dayton, OH, USA.
5. CSM Friction and Wear Testing Machine. Manufacturer: Tribometer Corporation, Murten, Switzerland.
6. BL-6157 Double-Rolling Mill. Manufacturer: Dongguan Baolun Precision Testing Instrument Co., Ltd., Dongguan, China.

These instruments serve a range of purposes, including material analysis, dispersion measurement, rubber processing performance analysis, and friction and wear testing.

### 2.2. Experimental Formula

The formula used in this experiment is shown in Table 1.

Natural Rubber (NR) has a density range of 0.92–0.93 g/cm^3^, an elongation rate of 800–1000%, and a tensile strength ranging from 2500 to 3500 psi. It is also available from Hainan Natural Rubber Industry Group Co., Ltd. (Wanning, China).

Styrene–Butadiene Rubber 9000 (BR9000), also known as polybutadiene rubber, possesses a density of 0.92 g/cm^3^, an elongation rate of 400–800%, and a tensile strength ranging from 2000 to 4000 psi. The size is 80 mm in diameter and 10 mm in thickness.

### 2.3. Performance Testing

#### 2.3.1. Rubber Processing Performance

The rubber processing performance was tested using the RPA 2000. The testing parameters for the RPA 2000 were set as follows: a multi-frequency, steady-state cooling with a scanning frequency of 0.01 Hz, a strain range of 0.28–40%, and a testing temperature of 60 °C.

Payne Effect: The Payne effect refers to the phenomenon of a rapid decrease in the dynamic modulus of filled rubber as the strain increases. The Payne effect reflects the distribution of the internal cross-linking network of the rubber. Generally, a more pronounced Payne effect indicates a denser internal cross-linking network and poorer filler dispersion.

#### 2.3.2. Tensile Tear Testing

To study the performance changes of compounded rubber after friction with different metals, the compounded rubber, after testing on the CSM machine, was subjected to rolling on the BL-6157 double-rolling mill.

Tensile and Tear Tests: Tensile and tear tests were conducted using the UT-2060 tensile testing machine. Dumbbell-shaped samples of vulcanized rubber were prepared, and tensile testing was performed with an extensometer. Five tests were conducted for each rubber sample, and the results were averaged to obtain the tensile data. Similarly, rectangular samples of vulcanized rubber without notches were used for tear testing. Five tests were conducted for each rubber sample, and the results were averaged to obtain the tear data.

#### 2.3.3. Vulcanization Properties Testing

Vulcanization properties were tested using the M-2000-AN rotorless vulcanization analyzer. Approximately 6 g of compounded rubber was placed in the rotorless rheometer cavity, and the instrument was set to a testing temperature of 150 °C with a testing time of 60 min. This resulted in the vulcanization curve and parameters such as ML, MH, TS1, TS2, and T90. Vulcanization testing not only provides information on the curing time of the rubber but also reflects the compounding effectiveness, making it an essential test in rubber processing.

#### 2.3.4. Rubber Dynamic Mechanical Properties Testing

Rubber dynamic mechanical properties were tested under the following conditions: temperature range −65 to 65 °C, heating rate of 2 °C/min, maximum dynamic load of 40 N, testing frequency of 10 Hz, and testing carried out using a dual cantilever test mode under liquid nitrogen protection [52,53,54].

#### 2.3.5. CSM Friction and Wear Testing

The CSM friction and wear testing machine can simulate pressure, temperature, and speed during the extrusion process. Therefore, for this experiment, the CSM machine was used to simulate the friction and wear behavior between compounded rubber and the extruder head metal. The wear on the metal head was observed to reflect the wear on the extruder head metal [55,56,57,58].

The flat-surfaced compounded rubber obtained from the BL-6157 double-rolling mill was cut into cylindrical rubber samples with a diameter of 100 mm and a thickness of 8 mm. After calibrating the CSM machine, its parameters were set to mimic the compounding process, with a pressure of 5 N and a speed of 70 r/min. Metal wear is challenging to observe over a short duration, so the friction time on the CSM machine was set to 60 min.

#### 2.3.6. Three-Dimensional Observation of Metal Surface

The Olympus LEXT OLS5000 3D scanning microscope was used to analyze the surface of the test samples before and after wear. This allowed for the observation of changes in the sample’s morphology and volume.

#### 2.3.7. Dispersion Testing

A new cross-section of vulcanized rubber was obtained using a cutter. The DisperGRADER dispersion analyzer was used to test this cross-section, directly providing dispersion values in accordance with ASTM D7723 standards [55,56,57,58].

## 3. Experimental Results

### 3.1. Dispersion Analysis

#### 3.1.1. Payne Effect

**Figure 1:**
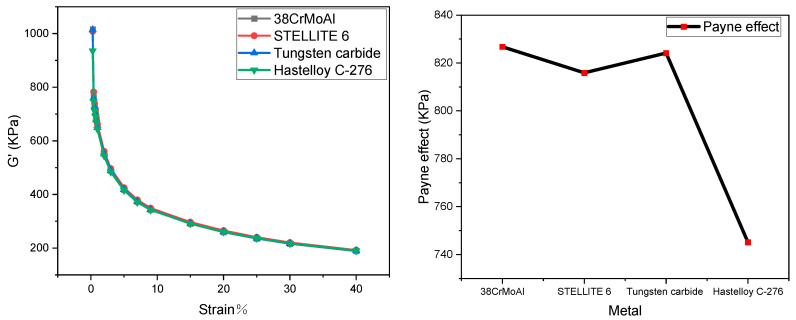
Payne Effect of NR/BR Composite Materials after Friction with Different Metals.

The stress–strain curves of NR/BR composite materials after friction with different metals are depicted in Figure 1. The Payne effect for NR/BR composite materials is presented in Table 2.

The data from Figure 1 have been extracted and presented in Table 2. As shown in Table 2, the NR/BR composite materials exhibit the highest Payne effect after friction with tungsten carbide alloy, while the Payne effect is the lowest after friction with Hastelloy C-276 alloy. The Payne effect of the same NR/BR composite material varies after friction with different metals, primarily depending on the wear resistance of the metal.

After friction between NR/BR composite materials and metal, the rubber undergoes abrasion due to abrasive wear caused by the rubber against the metal. As a result, tiny metal particles on the metal surface are worn away. These tiny metal particles become integrated into the compounded rubber, affecting the dispersion of fillers. Therefore, the quantity of tiny metal particles lost after friction with NR/BR composite materials differs based on the wear resistance of the metal. Compared to NR/BR composite materials after friction with Hastelloy C-276 alloy, the rubber Payne effect increased by 4.4% after friction with 38CrMoAl, 3.2% after friction with STELLITE 6, and 4.6% after friction with tungsten carbide. In general, the higher the quantity of tiny metal particles lost, the more severe the hindrance of the dispersion of SiO_2_ in the NR/BR composite materials by these tiny metal particles. Hence, from the Payne effect, it can be observed that NR/BR composite materials, after friction with tungsten carbide alloy, contain the highest quantity of tiny metal particles, while those after friction with Hastelloy C-276 alloy contain the lowest quantity of tiny metal particles. Moreover, the difference in the Payne effect of NR/BR composite materials after friction with the four metals is relatively small, which aligns with practical production conditions.

#### 3.1.2. Dispersion Image

**Figure 2:**
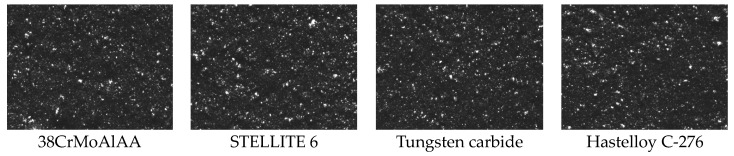
Dispersion Images of NR/BR Composite Materials after Friction with Different Metals.

From Figure 2 and Table 3, it is evident that the dispersion of NR/BR composite materials is highest after friction with Hastelloy C-276 alloy and lowest after friction with tungsten carbide alloy. Compared to NR/BR composite materials after friction with Hastelloy C-276 alloy, the rubber dispersion decreased by 9.4% after friction with 38CrMoAl, 4.7% after friction with STELLITE 6, and 9.8% after friction with tungsten carbide. This observation corresponds with the Payne effect of NR/BR composite materials.

### 3.2. Tensile Tear Performance

**Figure 3:**
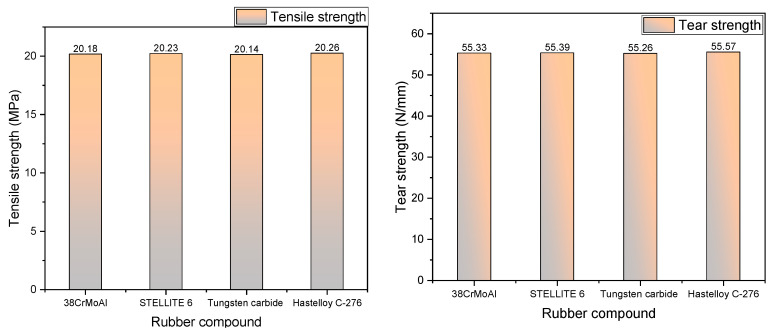
Tensile strength and tearing strength.

As depicted in Figure 3, the tensile strength and tear strength of NR/BR composite materials after friction with different metals exhibit a consistent trend. Compared to NR/BR composite materials after friction with Hastelloy C-276 alloy, the rubber tensile strength decreased by 0.39% after friction with 38CrMoAl, 0.15% after friction with STELLITE 6, and 0.59% after friction with tungsten carbide. The variations in tensile and tear strength of the same NR/BR composite material after friction with different metals are associated with the presence of tiny metal particles during the friction process. The better wear resistance of the metal leads to lower metal abrasion during friction, resulting in fewer tiny metal particles within the NR/BR composite material. Consequently, the changes in the tensile strength and tear strength of the NR/BR composite material are minimal. After friction with Hastelloy C-276 alloy, the NR/BR composite material exhibits the highest tensile strength and tear strength. This indicates that Hastelloy C-276 alloy causes less metal abrasion, resulting in fewer tiny metal particles within the NR/BR composite material. Thus, the impact of Hastelloy C-276 alloy on the performance of NR/BR composite materials is minimal.

### 3.3. Dynamic Mechanical Properties

**Figure 4:**
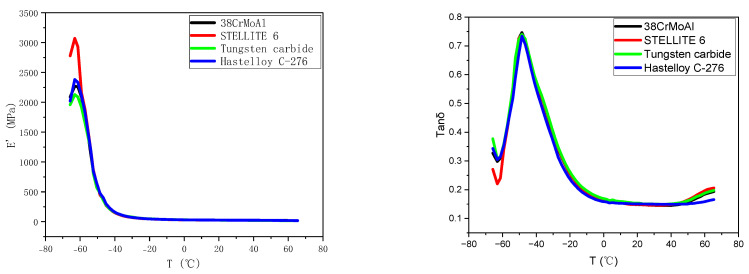
DMA Test Curves.

Figure 4 depicts the curves of E’ and Tanδ for NR/BR composite materials after friction with different metals as a function of temperature. In the rubber industry, the temperature corresponding to the maximum value of the Tanδ curve is commonly used to represent the glass transition temperature (TG), which can reflect the low-temperature resistance of NR/BR composite materials. Tanδ at different temperatures is typically employed to assess the dynamic mechanical properties of rubber, with Tanδ at 0 °C predicting the material’s resistance to wet skid, while values at 40 °C and 60 °C are indicative of rolling resistance. The temperature corresponding to the maximum value of the Tanδ curve is generally considered the glass transition temperature of the polymer.

Figure 4 illustrates the impact on the loss factor (tanδ) and E’. The data from Figure 4 are extracted and presented in Table 4. From Table 4, it can be observed that the glass transition temperature of NR/BR composite materials after friction with Hastelloy C-276 alloy is the lowest, while it is the highest after friction with tungsten carbide alloy. However, the temperature difference between these values is relatively small. This suggests that the NR/BR composite material exhibits the best low-temperature resistance after friction with Hastelloy C-276 alloy but slightly poorer performance after friction with the other alloys.

Additionally, based on the data in Table 4, it can be inferred that the NR/BR composite material has the best resistance to wet skid after friction with Hastelloy C-276 alloy, while the materials exhibit gradually decreasing resistance to wet skid after friction with STELLITE 6 alloy, 38CrMoAlA, and tungsten carbide alloy. The tanδ values at 40 °C and 60 °C indicate that the rolling resistance of the NR/BR composite material is the lowest after friction with Hastelloy C-276 alloy and gradually increases after friction with STELLITE 6 alloy, 38CrMoAlA, and tungsten carbide alloy. Compared to NR/BR composite materials after friction with Hastelloy C-276 alloy, the rubber rolling resistance increased by 18.1% after friction with 38CrMoAl, 16% after friction with STELLITE 6, and 23.4% after friction with tungsten carbide. This behavior is related to the presence of tiny metal particles in the compound rubber. These metal particles can increase the friction between the NR/BR composite material and the ground, thereby increasing rolling resistance. At the same time, the presence of tiny metal particles hinders the dispersion of SiO_2_ particles in the compound rubber. As a result, the more tiny metal particles there are, the more agglomerates of SiO_2_ particles there are in the compound rubber, reducing the material’s resistance to wet skid.

### 3.4. CSM Friction and Wear Experiment

#### 3.4.1. Average Friction Coefficient

**Figure 5:**
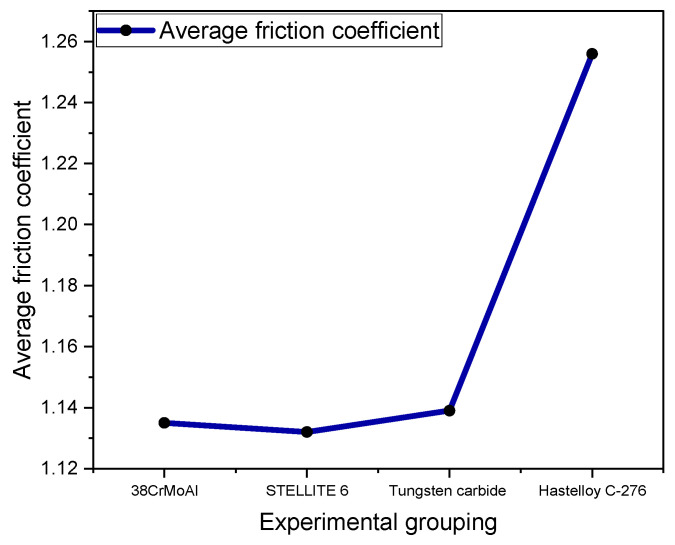
Average Friction Coefficient.

Figure 5 shows the average friction coefficient measured by CSM experiments.

Figure 5 displays the average coefficient of friction measured in the CSM experiment. The coefficient of friction is primarily related to the dispersion of SiO_2_ particles within the NR/BR composite material and is also linked to the presence of tiny metal particles. Generally, the better the dispersion of SiO_2_ particles and the fewer the tiny metal particles, the lower the coefficient of friction exhibited by the NR/BR composite material. The average coefficient of friction differs when the same NR/BR composite material is subjected to friction with different metals. This primarily depends on the wear resistance of the metal.

After friction between the NR/BR composite material and the metal, the rubber generates abrasive wear on the metal surface, causing the removal of tiny metal particles. These tiny metal particles become embedded in the mixed rubber, affecting the dispersion of the fillers. Therefore, owing to varying wear resistance in different metals, the number of tiny metal particles lost after friction with the NR/BR composite material varies. Generally, the greater the number of tiny metal particles lost, the more significantly they hinder the dispersion of SiO_2_ within the NR/BR composite material. Compared to the friction coefficient between Hastelloy C-276 alloy and NR/BR composite materials, the friction coefficient increased by 3.5% during the friction process with 38CrMoAl, 2.8% during the friction process with STELLITE 6, and 4.3% during the friction process with tungsten carbide. From Figure 5, it can be observed that the NR/BR composite material subjected to friction with tungsten carbide alloy exhibits the highest number of tiny metal particles within, while the NR/BR composite material subjected to friction with Hastelloy C-276 alloy has the fewest tiny metal particles.

#### 3.4.2. Three-Dimensional Morphology

**Figure 6:**
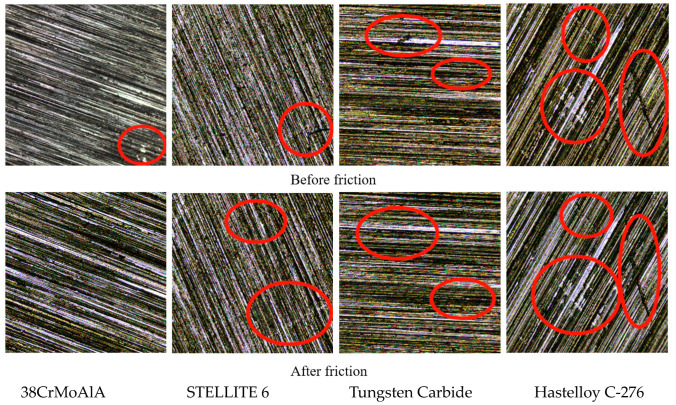
Metal Surface Morphology (×10).

**Figure 7:**
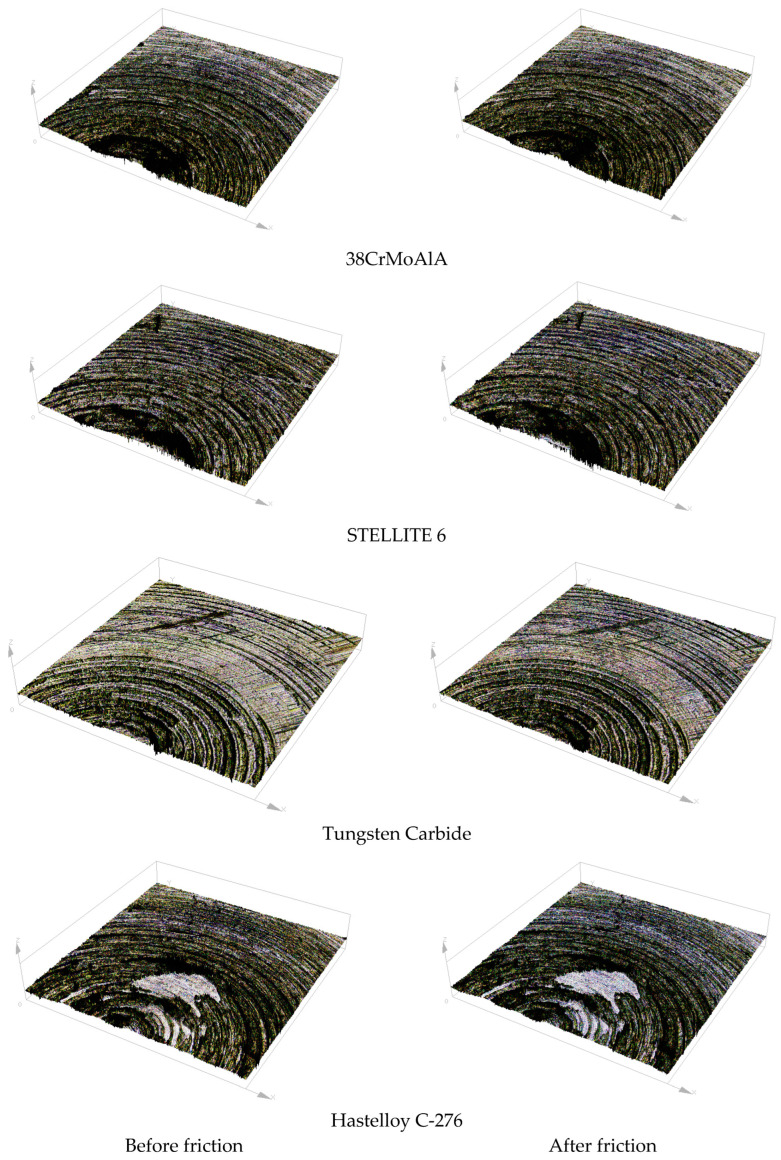
Three-Dimensional Morphology of Metal Surface Before and After Friction.

Figure 6 shows the surface morphology of the metal grinding head before and after friction. Figure 7 shows the three-dimensional morphology of the metal grinding head surface. As can be seen from Figure 6 and Figure 7, many scratches appeared on the surfaces of the four metals after friction. Among them, the pits on the surface of tungsten carbide alloy expanded significantly after friction.

#### 3.4.3. Height Profile

**Figure 8:**
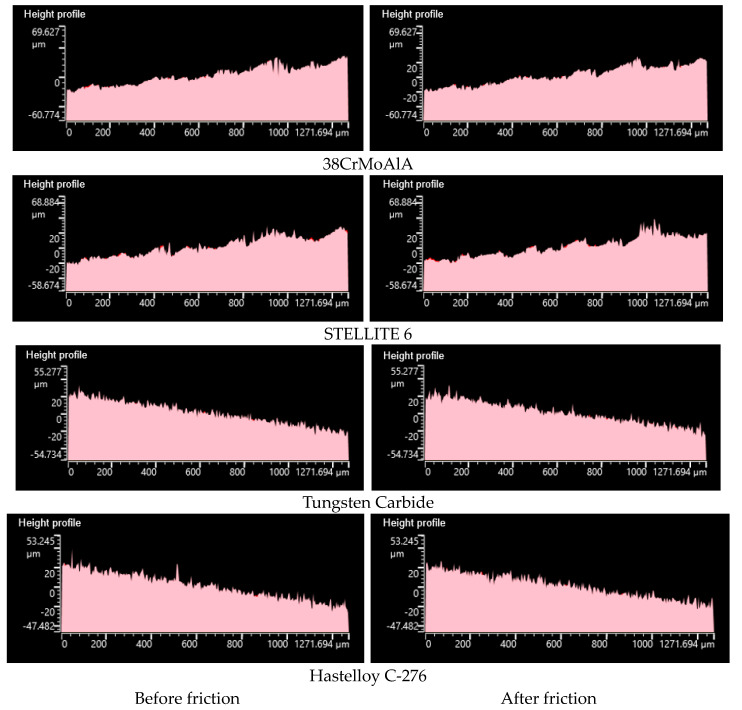
Profilometer Images of Different Metals Before and After Friction with NR/BR Composite Materials.

Figure 8 presents the height profiles of different metals before and after friction with NR/BR composite materials. It is evident that after friction, Hastelloy C-276 alloy exhibits the least change in the metal surface’s height profile, indicating its superior wear resistance. STELLITE 6 alloy and 38CrMoAlA also display minimal changes in the metal’s height profile after friction, with only a few peak contours being flattened. This suggests relatively good wear resistance for STELLITE 6 alloy and 38CrMoAlA. In contrast, tungsten carbide alloy experiences significant changes in the metal’s height profile, with numerous peak contours being flattened and a noticeable difference in height profiles before and after friction. This indicates that tungsten carbide alloy has relatively poorer wear resistance compared to the other four alloys.

#### 3.4.4. Metal Volume Change

**Figure 9:**
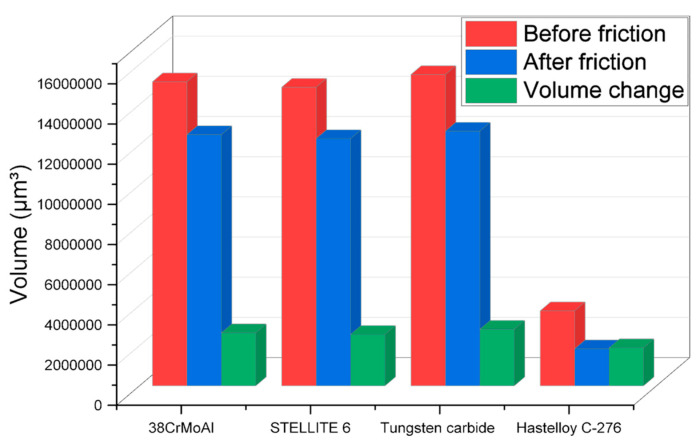
Changes in Metal Volume Before and After Friction with NR/BR Composite Materials.

The changes in metal volume before and after friction with NR/BR composite materials for different metals are displayed in Figure 9.

Figure 9 reveals that after friction with NR/BR composite materials, Hastelloy C-276 alloy experiences significantly lower metal wear compared to the other three metals. Compared to the wear volume between Hastelloy C-276 alloy and NR/BR composite materials, the wear volume increased by 39.3% during the friction process with 38CrMoAl, 45.3% after friction with STELLITE 6, and 48.9% after friction with tungsten carbide. The difference in metal wear between STELLITE 6 alloy and 38CrMoAlA is relatively small, while tungsten carbide alloy exhibits the highest metal wear after friction. From a wear perspective, Hastelloy C-276 alloy demonstrates far superior wear resistance compared to the other three metals.

#### 3.4.5. Surface Roughness

**Figure 10:**
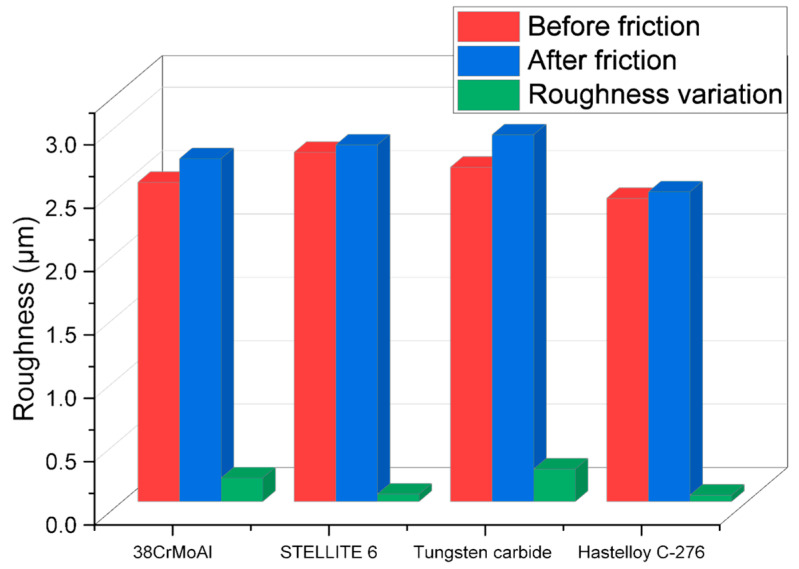
Variation in Surface Roughness of Different Metals Before and After Friction with NR/BR Composite Materials.

The changes in metal surface roughness before and after friction with NR/BR composite materials for different metals are depicted in Figure 10.

As observed in Figure 10, Hastelloy C-276 alloy and STELLITE 6 alloy exhibit minimal changes in surface roughness after friction with NR/BR composite materials, while 38CrMoAlA and tungsten carbide alloy show significant alterations in metal surface roughness following friction with NR/BR composite materials. Particularly, tungsten carbide alloy experiences the most substantial increase in surface roughness after friction. The variations in metal surface roughness are primarily attributed to the aggregation of SiO_2_ particles and the presence of minute metal particles within the composite. Notably, metals with superior wear resistance have fewer embedded minute metal particles in the NR/BR composite, resulting in less hindrance to the dispersion of SiO_2_ particles. Consequently, a lower number of SiO_2_ particle aggregations in the NR/BR composite material corresponds to reduced changes in surface roughness following friction. This suggests that the extent of changes in metal surface roughness is directly proportional to the wear resistance of the metal. Figure 10 provides a visual representation of the wear resistance of the four alloys studied.

## 4. Conclusions

This study meticulously compares the wear resistance of four different metal materials commonly utilized in extrusion molding machine heads. Rooted in extensive experimental analysis, the research offers data-driven insights crucial for making informed decisions when selecting metallic materials for these machine heads. The results highlight significant variations in rubber performance after friction with different metals. In comparison to friction with NR/BR composite materials and Hastelloy C-276 alloy, the rubber Payne effect increases by 4.4% with 38CrMoAl, 3.2% with STELLITE 6, and 4.6% with tungsten carbide. Following friction with these metals, rubber dispersibility decreases by 9.4%, 4.7%, and 9.8%, respectively, while rolling resistance increases by 18.1%, 16%, and 23.4%. In the friction process, coefficients increase by 3.5% with 38CrMoAl, 2.8% with STELLITE 6, and 4.3% with tungsten carbide compared to the friction coefficients of Hastelloy C-276 alloy and NR/BR composite materials. Moreover, wear volume increases by 39.3%, 45.3%, and 48.9% with 38CrMoAl, STELLITE 6, and tungsten carbide, respectively. Opting for Hastelloy C-276 alloy demonstrates optimal dispersibility, the highest tensile tearing performance, excellent anti-wet sliding capabilities, and the lowest rolling resistance in NR/BR composite materials. However, selecting the other three alloys results in varying degrees of reduction in the physical–mechanical properties of NR/BR composite materials. This reduction correlates with the wear resistance of the metal, where higher wear resistance leads to fewer embedded micro-metal particles, consequently enhancing the performance of NR/BR composite materials. In summary, the study confirms that Hastelloy C-276 alloy exhibits outstanding wear resistance, while 38CrMoAlA and tungsten carbide alloys show comparatively poorer wear resistance. These findings provide valuable insights for judiciously selecting metallic materials to achieve optimal performance in extrusion molding machine heads.

## Figures and Tables

**Table 1:** Experimental formula.

Raw Material	C1
NR	40
N330	25
SiO_2_	25
4020	5
ZnO	3
SAD	2
TESPT	6
DPG	0.18
S	2.8
CZ	1.8

**Table 2:** Payne Effect of NR/BR Composite Materials after Friction with Different Metals.

Metal	38CrMoAlA	STELLITE 6	Tungsten Carbide	Hastelloy C-276
Payne effect	826.76	815.89	824.15	745.12

**Table 3:** Dispersion Values of NR/BR Composite Materials after Friction with Different Metals.

NR/BR Composites	38CrMoAlA	STELLITE 6	Tungsten Carbide	Hastelloy C-276
Dispersion	5.87	6.09	5.85	6.16

**Table 4:** Glass Transition Temperature (TG) and Tanδ.

DMA	38CrMoAlA	STELLITE 6	Tungsten Carbide	Hastelloy C-276
TG (°C)	−47.0562	−47.1293	−46.6551	−47.2175
0 °C tanδ	0.15034	0.152085	0.145559	0.156582
40 °C tanδ	0.135945	0.134021	0.136972	0.131308
60 °C tanδ	0.172737	0.169484	0.181068	0.14421

## Data Availability

Data are contained within the article.
